# Polyomavirus BK and prostate cancer: an unworthy scientific effort?

**DOI:** 10.18632/oncoscience.32

**Published:** 2014-04-30

**Authors:** Serena Delbue, Pasquale Ferrante, Maurizio Provenzano

**Affiliations:** ^1^ Department of Biomedical, Surgical and Dental Sciences, University of Milano, Italy; ^2^ Oncology Unit, Division of Urology, University Hospital of Zurich, Switzerland

**Keywords:** Polyomavirus BK, BKV-DNA detection, prostate cancer, meta-analysis

## Abstract

The Polyomavirus BK (BKV) has been proposed to be one of the possible co-factors in the genesis of prostate cancer (PCa) but, so far, the only convincing suggestion is the hypothesis of a “hit and run” carcinogenic mechanism induced by the virus at early stages of this disease. To support this hypothesis we conducted an updated systematic review on previous studies regarding the association between BKV and PCa, in order to interpret the contrasting results and to explore whether there might be a significant virus-disease link. This updated analysis provides evidence for a significant link between BKV expression and PCa development, particularly between the BKV infection and the cancer risk. Forthcoming scientific efforts that take cue from this study might overcome the atavistic and fruitless debate regarding the BKV-PCa association.

## Polyomavirus BK and prostate cancer; an unsolved dilemma

Prostate cancer (PCa) is the third most common cause of morbidity and the fourth leading cause of cancer death in western countries [1], but it is becoming increasingly more relevant worldwide due to higher life expectancy and refinement of diagnostic procedures [2, 3]. Among other consistent risk factors [4-6], the pathogenesis of this malignancy reflects chronic inflammatory states and the proliferative inflammatory atrophy (PIA) has been postulated to be the key transition step toward overt PCa [7]. Infectious agents have been ranked among inflammatory-related factors that are important for PCa onset [8]. This also includes viruses that presumably play a causative role in PIA development [9]. However, virus involvement in prostate carcinogenesis remains to be demonstrated [10]. Human Polyomavirus BK (BKV) is a circular double stranded DNA virus that belongs to the Polyomaviridae family [11]. It establishes a life-long persistent asymptomatic infection in the urinary tract latently residing in the urothelium [12]. Both human and cellular immune responses mounted against capsid antigens patrol the viral activity in immumocompetent individuals but the balance between immune defence and viral fitness is mainly due to cellular immune responses when reactivations occur [13, 14], particularly at sites of smoldering infections [15]. However, when the immune system is compromised following an ablative therapy before organ transplantation, after HIV associated immunosuppression or pharmacologic immunosuppression, this immune balance is lost and the viral reactivation leads to a productive infection in permissive cells with the release of new virions in the peripheral blood (viremia) and their shedding in the urine (viruria) [16, 17]. The increase in rate and level of BKV replication might lead to severe diseases at the anatomical site of relevance, such as the hemorrhagic cystitis in bladder and/or the polyomavirus-associated nephropathy (PVAN) in kidney, which is the principal cause of the transplant rejection of the organ [18, 19]. In contrast, the viral entry in non-permissive cells can lead to an oncogenic transformation as a consequence of an abortive infection [20].

Although several oncogenic viruses have already been linked to human malignancies [21], such as papillomavirus (HPV) to cervical and anogenital cancer [22], Epstein-Barr virus (EBV) to Burkitt's lymphoma [23] and nasopharyngeal carcinoma [24, 25], the confirmation of the effects of polyomaviruses in the genesis of human cancers, except for Merkel Cell polyomavirus and Merkel Cell carcinoma [26], has proven more difficult. This in turn rendered the acceptance of a causal role of these viruses in the etiology of human cancers much harder. The oncogenic activity of polyomaviruses has been documented *in vitro* in cell lines [27] and in hamsters [28]. It is exerted through the main regulatory protein L-Tag which binds products of tumor suppressor genes (pRb family, p53) thus interfering with the strategic checkpoints of the cell cycle of infected cells [29]. In addition, the p53/L-Tag complex binds and activates the insulin-like growth factor 1 (IGF-1) thus potentiating the cancer transformation of infected cells [30]. Indeed, it has lately been proposed that the main hallmark of the polyomavirus involvement in cancer development might be the presence of the wt-p53/L-Tag complexes in the cytoplasm of transformed cells. This complex appears after the binding of L-Tag to the wt-p53 in the nucleous of infected cells and the sequestration of the suppressor protein in their cytoplasm [31]. This might suggest the ranking of BKV L-Tag among the wider range of tumor inducers as a potential co-factor for PCa development [9]. Despite evidence of DNA detection and expression of viral gene products in pre-cancerous/cancerous lesions of the prostate, discrepancies between polyomavirus BK infection of the organ and the onset of human prostate cancer are still ruling the scientific discussion [32]. Is this investigation thus worth the efforts if there might not be any solid scientific ground?

## A narrow borderline between BKV behavior as a co-factor or bystander: the “hit and run” hypothesis

**Figure 1:**
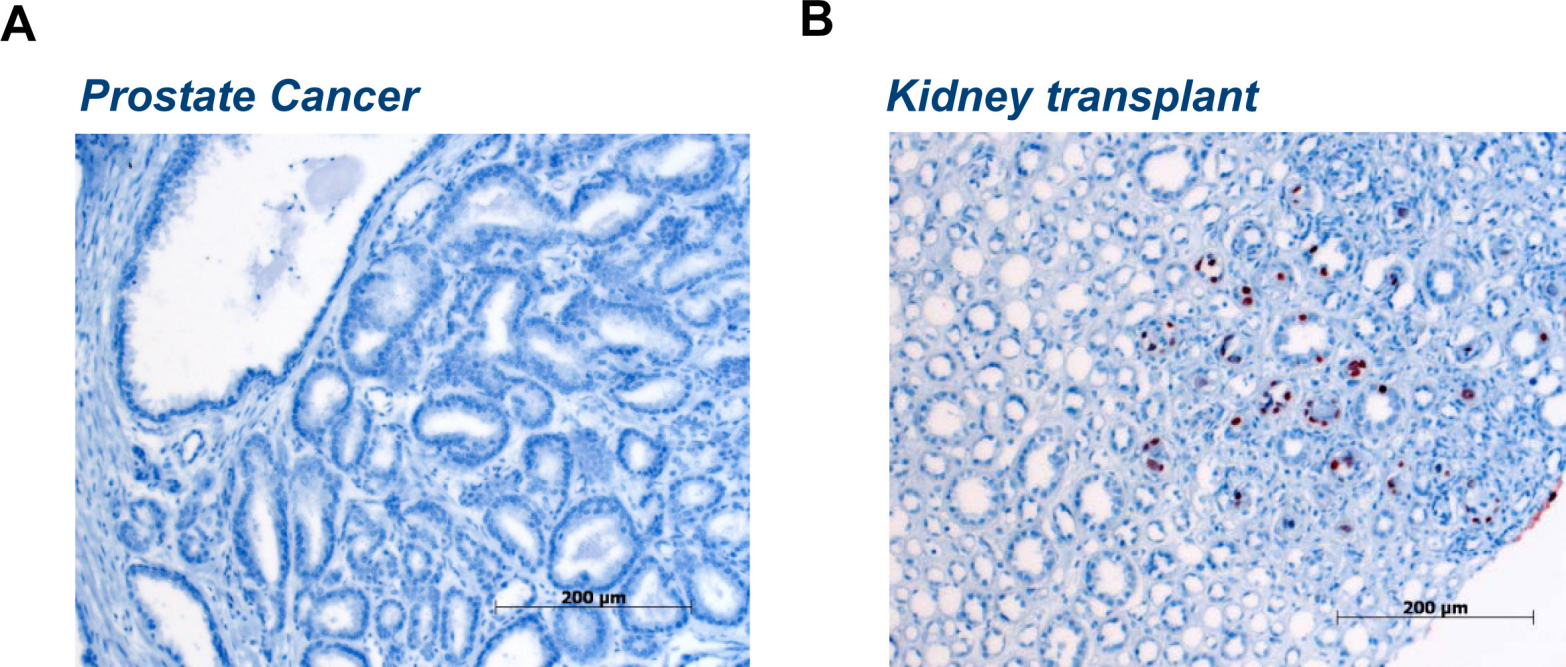
Discrepancies between BKV L-Tag DNA detection and IHC in tumor specimens A) A representative PCa tissue specimen, among those tested for molecular detection of BKV L-Tag DNA in Sais et al. [57], shows an evident lack of IHC staining for BKV L-Tag, as compared to B) a tissue specimen from a kidney transplant with virus reactivation (unpublished data provided by the authors).

Plenty of work is required to discover the molecular mechanism underlying the viral oncogenic activity [33] and to understand how oncogenic viruses interfere with and orchestrate the tumor microenvironment [34, 35]. The “hit and run hypothesis” is the most valid proposition to justify a co-factorial role of BKV in PCa onset and progression [9]. It has been introduced by Skinner in 1976 [36] and re-proposed by Galloway in 1983 [37] to stress the oncogenic potential of human herpes viruses (HSV) in cervical cancer and recently by Stevenson to provide experimental support [38]. The hypothesis also helps to explain the disparity between the gene expression of BKV L-Tag in prostatic tumor specimens and its rare expression at protein level in same specimens (Fig 1). It thus seems that the ability of polyomavirus to interfere with the cell cycle could induce the infected cells to reach the critical point of no return during oncogenic transformation [39]. Once the cell cycle is manipulated and the sequestration of p53 is accomplished by L-Tag in the context of an abortive infection, accumulation of gene mutations in the infected cells might lead to transformation without a continuous “support” of viral components [40, 41]. The activity of the virus paves the way for tumorigenic transformation at early stages of PCa and the presence of viral fitness in the tumor cells is no longer necessary to charge the tumor causality to the virus itself [38, 42]. Nonetheless, the “hit and run” theory is hard to be sustained experimentally due to the obvious difficulty in providing evidence for a resolved infection. In overt cancer, it will lead to a drastic reduction of sensitivity for tests detecting viral genes due to the complete disappearance of the virus in tumor cells [38]. To support the “hit and run” theory, in the present study we assessed a cumulative prevalence of BKV in PCa. We calculated the risk of cancer development with BKV infection on previous studies regarding the association between BKV and PCa in order to interpret the contrasting results and to explore whether there might be a significant virus-disease link.

**Figure 2:**
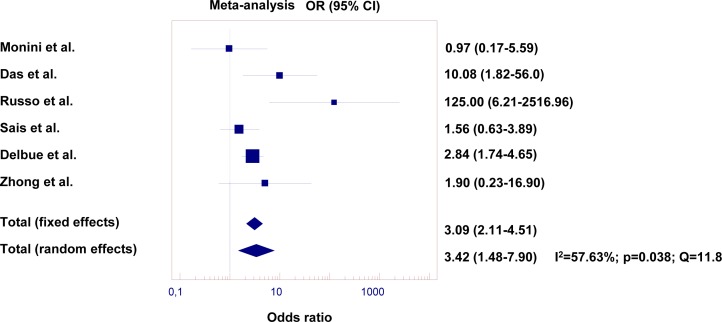
Risk of PCa with BKV infection in tumor samples compared with non-tumor prostate samples The PCa risk with the presence of BKV infection was evaluated by a pooled odds ratio (OR). For measure of inter-study heterogeneity, we used the Cochran's Q test. Fixed effects (Mantel-Haenszel) and random effects (DerSimonian and Laird) models were performed based on the percentage of variation across studies (I^2^ statistic). All p-values <0.05 were considered statistically significant.

## BKV-DNA expression in cancer specimens: original reports against specimens' adjustment

According to new features of early pre-cancerous lesions in their progression to overt PCa [43], we performed an adjustment in the case group including also both non-neoplastic/precancerous and early-stage cancer lesions from cancer bearing prostate, which originally were attributed to the control group. This “specimens' adjustment” allowed us to extend the search for virus expression into lesions at very early stages of PCa development. Consequently, in the case-control study we considered a “case” each tumor specimen obtained from PCa patients enrolled in each study and a “control” each specimen originating from non PCa patients, such as benign prostatic hyperplasia (BPH) patients or healthy tissues from other patients. We then particularly compared a standard analysis to a specific one by adding the adjustment of tissue samples. With this updated analysis we were aiming at confirming the co-factorial activity of polyomavirus BK in prostate cancer by categorizing its expression in tumor lesions into marginal, moderate or substantial for risk of PCa development. A limited Medline search for the keywords “BK virus”, “prostatic” and “prostate cancer” identified twenty-five papers. Fifteen out of the twenty-five articles met the criteria by using the molecular-based techniques for the detection of BKV DNA or proteins in tissues. The other ten were excluded for the following reasons: reviews (n=3) [44-46], serological studies (n=3) [47-49], case report (n=1) [50], comment (n=1) [51], cell line study (n=1) [52] or publication in language other than English (n=1) [53]. Out of the fifteen articles included in the analysis, eight were carried out in Europe (Italy [54-56], Switzerland [57], UK [58], Sweden [59], Greece [60], Germany [61]), five in USA [9, 31, 62-64], one in Mexico [65] and one in Japan [66]. Due to lack of information if tests were performed considering one specimen as one patient (i.e. multiple tissue collection from each enrolled patient), we included each tumor tissue (case) or normal tissue (control) specimen analyzed for BKV-DNA expression in our statistic. Statistical analysis was performed with MedCalc software (v12.7.7). Estimations (95% CI) of single prevalence and pooled prevalence were determined by meta-analysis of either original reports or after adding the “specimens' adjustment”.

Based on the original reports, a total of 1106 cancers, ranging from 7 to 328 (mean value=74; n=15), and 1068 controls, ranging from 11 to 385 (mean value 97; n=11) were analyzed. The prevalence of BKV was significantly higher in cancer tissues than in the control tissues (p<0.0001). Indeed, the range was 0% to 84.6%, with an overall value of 13.2% (95% CI: 11.2%-15.9%) in cancers, and 0% to 57.9% with an overall value of 5.6% (95% CI: 4.2%-7.0%) in controls (Table 1). Three groups collected as control specimens non neoplastic prostate tissues from areas surrounding cancer lesions, such as inflammatory/atrophic specimens adjacent to or in transition to prostate adenocarcinoma and early-stage cancer lesions, such as high-grade (HG)-PIN [62-64]. To perform the adjustment, we included these specimens in the case group (1106 cancers) thereby increasing the total number of cases to 1399 (range 7-328, mean=93.3; n=15) while the number of controls decreased to 775 (range 12-385, mean=96.9; n=8) after adjustment. The prevalence values varied from 0% to 84.6%, with an overall value of 10.8% (95% CI 9.3%-12.9%) among cases and from 0% to 57.9% with an overall value of 6.9% (95% CI 5.1%-8.7%) among the controls (Table 1). Overall, BKV-DNA was detected in about 10% of prostate tissue specimens tested (206/2174) in 80% of the studies (12/15) analyzed. Although the prevalence of BKV-DNA detection was obviously reduced in the case group after the adjustment, however not enough to be significant (original *vs* adjusted analysis p=0.08), the modest increase of percentage of BKV-DNA positive lesions in the adjusted group (146 to 152 = 4%) is noteworthy since the significant association between viral expression and cancer observed analyzing the original reports is maintained when including as “cases” all specimens from cancer bearing prostate tested (p<0.01).

**Table 1:** Studies selected for the analysis by a limited Medline. BKV-DNA prevalence in case and control group according to “specimens' adjustment”

	Case group						Control group					
Studies	Case tested	Case positive	prevalence (%)	Case after adj	Case pos after adj	prevalence after adj(%)	Control tested	control positive	prevalence (%)	control after adj	control pos after adj	Prevalence after adj (%)
Monini	7	4	57.1	7	4	57.1	19	11	57.9	19	11	57.9
Zambrano	7	2	28.6	18	3	16.7	11	1	9.1	-	-	
Das	21	15	71.4	21	15	71.4	-	-		-	-	
Bergh	171	0	0.0	171	0	0.0	181	0	0.0	181	0	0.0
Lau	30	2	6.7	99	7	7.1	69	5	7.2	-	-	
Sfanos	113	1	0.9	326	1	0.3	213	0	0.0	-	-	
Balis	42	8	19.0	42	8	19.0	-	-		-	-	
Das	14	11	78.6	14	11	78.6	15	4	26.7	15	4	26.7
Russo	26	22	84.6	26	22	84.6	12	0	0.0	12	0	0.0
Martinez-Fierro	55	0	0.0	55	0	0.0	75	0		75	0	0.0
Akgul	85	1	1.2	85	1	1.2	-	-		-	-	
Groom	100	0	0.0	100	0	0.0	-	-		-	-	
Sais	43	18	41.9	43	18	41.9	38	12	31.6	38	12	31.6
Dehde	328	56	17.1	328	56	17.1	385	26	6.8	385	26	6.8
Zhong	64	6	9.4	64	6	9.4	50	1	2.0	50	1	2.0
TOT	1106	146	13.2	1399	152	10.9	1068	60	5.6	775	54	6.9
	95% CI 11.2-15.9					95% CI 4.2-7.0		95% CI 9.3-12.9				95% CI 5.1-8.7

**BKV-DNA prevalence in case and control groups according to original reports and the adjustments of tissue specimens tested**.

**In bold: studies that used tissues from cancer bearing prostate as controls**

## BKV-DNA detection rate among techniques used and types of specimen tested

The methods for molecular-based testing for BKV-DNA detection were: a) the gene assay methods using nested-PCR (n=9), thereof 7 with type-specific primers for L-Tag (n=6) or VP1 (n=1) and 2 with broad spectrum primers; b) regular PCR with broad-spectrum primers for BKV and JCV VP1 genes (n=1); c) quantitative real time PCR with specific primers and probe (qRT-PCR; n=4) targeting either L-Tag or VP1 genes or using a kit without any specification. The detection rate for BKV-DNA in tumor tissues by qRT-PCR (482; 44.8%) was significantly higher than the rate obtained by both nested and regular PCR (594; 55.2%) either before (20.1%; 95% CI 16.5%-23.7% *vs* 7.9%; 95% CI 5.2%-9.0%, p<0.0001) or after the adjustment (qRT-PCR n=482; 20.1%; 95% CI 16.5%-23.7% *vs* regular PCR n=818; 6.0%; 95% CI 4.5%-7.5%, p<0.0001) (Table 2).

**Table 2:** BKV-DNA prevalence in PCa according to techniques used and types of specimen tested with and without adjustment

Categories	No of studies	No of tissues	Prevalence (%)	95% CI	No of tissues after adjustment	Prevalence (%) after adjustment	95% CI
Total	**15**	**1106**	13.2	11.2-15.9	**1399**	10.8	9.3-12.9
**Technique (total)**	**14**	**1076**			**1300**		
PCR/nested PCR	10	594	7.9	5.2-9.0	818	6.0	4.5-7.5
qRT-PCR	4	482	20.1	16.5-23.7	482	20.1	16.5-23.7
**Type of specimen (total)**	13	**1057**			**1350**		
Paraffin-embedded tissue	8	490	14.1	16.3-22.5	559	13.2	10.4-16.0
Fresh tissue	5	576	11.5	8.8-14.0	791	8.3	6.4-10.2

Two additional laboratory methods were employed to analyze BKV expression in tissue specimens. However, due to the limited number of specimens tested the calculation is reported only for completeness of information (data not shown). The “in situ hybridization” (ISH) assay employing a BKV specific probe was carried out in three studies (cases, n=79) [9, 31, 62], showing a strong prevalence of 26.6% (95% CI 11.2%-28.2%). For the detection of BKV proteins, four groups [9, 31, 56, 62] employed immunohistochemistry (IHC) assays (cases, n=105). In particular, Das et al [9] used an anti-VP1 Antibody (P5G6BKV9VP1) while the other groups identified the L-Tag protein using the antibody pAB416 that cross reacts with SV40 and JCV. BKV proteins were significantly more expressed in tumor tissues (40%; 95% CI 30.6%-49.4%) than in normal tissues (5.6%; 95% CI 0.12%-7.1%; p<0.0001).

Thirteen out of 15 studies mention the type of specimens analyzed in their “material and methods” section. Thus, five studies used fresh or frozen sections (F/F n=576, 53.6%), whereas eight studies used formalin fixed, paraffin embedded tissues (FFPE n=490; 46.4%). The efficiency at detecting BKV-DNA in FFPE tissue was equal to frozen sections since the detection of BKV-DNA in FFPE tissue was 14.1% while in frozen section was 11.4% (p=0.23). Conversely, the difference was highly significant when comparing FFPE (n=559; 41.4%) to frozen sections (n=791; 58.6%) after the adjustment (13.2% *vs* 8.3%; p=0.003) (Table 2). The adjustment confirmed that qRT-PCR is the best gene assay method for BKV DNA detection in tissues and it supports the use of FFPE tissue specimens for molecular-based testing. The latter is of importance since, in addition to the shortage of prostate cancer tissues owing to increasing new therapeutic approaches, such as active surveillance [67], fresh/frozen specimens are also rarely collected for research purpose, due to their prioritized use for diagnostic purposes and the small dimensions of the tumor at first diagnosis [68].

## A link between BKV expression and PCa cancer risk supports future investigations

Six case-control studies with 482 case tissues from PCa patients and 519 control tissues collected from patients with BPH were statistically compared for the estimation of BKV infection and prostate cancer risk. Two studies were automatically excluded after data pooling [59, 65], because of the absence of BKV expression in both cases and controls.

The prevalence of BKV was 16.5% among the tissues from PCa patients (95% CI 13.8%-19.2%) and 7.0% among the control tissues from BPH patients (95% CI 5.2%-8.8%; p<0.0001). According to the results of inter-study heterogeneity (I2 >50%), the random-effect model was used to evaluate the pooled OR. Overall, there was a significantly increased prostate cancer risk with the presence of BKV infection compared with the BPH controls (OR=3.4, 95% CI: 1.5-7.9, p=0.04; Fig. 1) based on an analysis assuming random models (I2 =57.6 Q=11.8). Adding studies (n=3) using pre-cancerous and early-stage cancer tissues as paired controls (case n=632, controls n=812), an overall prostate cancer risk with the presence of BKV infection (OR=2.94, 95% CI 1.51-5.73, p=0.09) was marginally maintained assuming random models (I2 =63.4 Q=13.7). Obviously, the case-control analysis after the specimens' adjustment was not performed due to the lack of control tissues from the three added studies. It is noteworthy that our analysis of the case-control study showed a significantly increased risk for PCa development only when the non-malignant control group consisted of BPH patients, while adding studies that used non neoplastic prostate tissues from PCa bearing patients as control reduced the risk of PCa development to a no longer significant value. This confirms that non-neoplastic prostate tissues adjacent to cancer lesions or cancer-surrounding areas cannot be considered genuine controls, thus justifying the use of specimens' adjustment in this analysis and future investigations.

This link might contribute greatly to clarifying the role of BKV in cancer, and raise the awareness that the virus plays a co-factorial role rather than just being a mere bystander in the development of the tumor.

## CONCLUSION

Prostate cancer is the most common malignancy affecting males and a clinically heterogeneous disease characterized by a variety of histological conditions that render the selection of pure prostatic adenocarcinoma from surrounding non neoplastic areas a difficult task [69]. In some studies used for this meta-analysis, the majority of positive results originated from diagnostic tests performed in inflammatory areas surrounding the cancer and/or atrophic areas at transition zones. In particular, inflammation is responsible for either predisposing the prostate to malignant transformation by carcinogens or for initiating and promoting cancer generation by itself [43, 70]. It has been postulated that BKV might be involved in the etiology of main precursor stages of prostate cancer, such as PIA, in areas where histological transitions between inflammation and cancer occur [9, 43]. Therefore, the search of BKV expression at very early stages of neoplastic progression, such as at PIA or PIN levels, is of crucial importance and adjacent tissue surrounding tumor areas or atrophic regions cannot be considered normal controls *per se* [71].

Our investigation differs from previous analyses in two important aspects. First it is updated to April 2014 including four papers released in 2012 and 2013 which matched our selection criteria. Three of these recently published papers found a positive correlation between the expression of BKV-DNA in cancer specimens and viral features [54, 57, 66]. Second, we adopted a novel strategy in grouping cases and controls. We particularly noted that the adjustment of tissue sampling by including areas being in contact with the cancer, or tissues picked in same cancer bearing organs, either confirmed or improved the statistical significance or even made it more powerful, but it never worsened the situation.

Our findings are thus consistent with a significant link between the BKV infection and the PCa risk. There is thus ample evidence for a potential co-factorial role of BKV in PCa. A confirmed association between the detection of BKV in cancer lesions, particularly in prostate cancer, and the malignancy could represent a first-step learning model on the causative role of polyomaviruses in cancer. This investigation is thus not an unworthy scientific effort, but rather deserves to be standardized in some ways. The gaps are mainly due to sampling collection schemes and selection of controls. Despite limited, this study might also induce new investigators to provide with new experimental support to understand the interactions between potential oncogenic viruses and the development of cancers.
